# Modified versus three-level technique of retroperitoneal laparoscopic adrenalectomy for all patients with adrenal lesions ≤ 6cm: a retrospective, case-controlled study

**DOI:** 10.3389/fendo.2024.1342240

**Published:** 2024-03-04

**Authors:** Minxiong Hu, Yangbiao Wu, Xiao Xu, Wenchao Cai, Jiangui Lin, Wanghai Cai, Honghong Pan, Zesong Yang, Liefu Ye, Jinfeng Wu

**Affiliations:** ^1^ Shengli Clinical Medical College of Fujian Medical University, Fuzhou, Fujian, China; ^2^ Department of Urology, Fujian Provincial Hospital, Fuzhou, Fujian, China; ^3^ The Graduate School of Fujian Medical University, Fuzhou, Fujian, China; ^4^ Department of Radiology, Fujian Provincial Hospital, Fuzhou, China; ^5^ Department of Urology, Zhangzhou Municipal Hospital, Zhangzhou, Fujian, China; ^6^ Department of Urology, Fujian Provincial Hospital South Branch, Fuzhou, Fujian, China

**Keywords:** adrenal gland, adrenal lesions, adrenalectomy, retroperitoneal laparoscopy, minimally invasive surgery, modified technique

## Abstract

**Objectives:**

The modified three-level technique for retroperitoneal laparoscopic adrenalectomy (RLA) has proven beneficial in the treatment of adrenal lesions in patients with BMI≥25 Kg/m^2^. This paper aims to summarize our institution’s seven-year experience using this technique for all patients with Adrenal Lesions ≤ 6cm.

**Patients and methods:**

Between January 2016 and December 2022. The patients underwent laparoscopic adrenal surgery were categorized into Zhang’s technique (ZT) (Three-level Technique) group and modified technique (MT) group. The fundamental characteristics and perioperative data were analyzed, with statistical significance set at p<0.05.

**Results:**

In total, 731 patients were stratified into two groups: ZT (n=448) and MT (n=283). Statistically significant distinctions were not detected between the two groups regarding sex, BMI, tumor location, tumor size, tumor type, or American Society of Anesthesiologists (ASA) score (p>0.05). The MT group demonstrated superior outcomes compared to the ZT group in terms of operative time, estimated blood loss, drainage volume, diet recovery time, complication rates, and postoperative hospitalization duration (p<0.05). 17 (4.34%) in the ZT group required unplanned adrenalectomy, while there was none in MT group (P<0.05).

**Conclusion:**

MT retroperitoneal laparoscopic adrenalectomy has demonstrated its benefits in the treatment of adrenal lesions across all patients with adrenal lesions ≤ 6cm, serving as a valuable point of reference for the surgical management of adrenal diseases.

**Patient summary:**

We have made modifications to the classic retroperitoneal laparoscopic adrenalectomy and achieved superior surgical outcomes, resulting in a procedure known as modified retroperitoneal laparoscopic adrenalectomy. This technique is suitable for both obese individuals and the general population with adrenal lesions ≤ 6cm.

## 1 Introduction

Adrenalectomy is the definitive treatment for multiple adrenal abnormalities (1). The safety of minimally invasive adrenalectomy can be achieved through two approaches: retroperitoneal laparoscopic adrenalectomy (RLA) and transperitoneal laparoscopic adrenalectomy (TLA) (2). with equivalent rates of major complications and mortality (1, 3, 4). Some studies suggested RLA tends to be performed for smaller tumors (3, 5). Nevertheless, when comparing RLA and TLA, RLA operations exhibit superior outcomes in terms of reduced surgery duration, decreased blood loss, alleviated postoperative pain, quicker recovery, improved cost-effectiveness, and eliminated risk of surgical access site herniation (2, 4). In addition, the surgical conversion rate of RLA is higher in the transperitoneal route (3).

Adrenal pathologies are mostly benign, making organ-preserving procedures attractive for many patients (6). RLA is safe, feasible, and has therapeutic results similar to TLA in patients with a non-hereditary hormonally active unilateral adrenal mass (7). Furthermore, RLA has gained significant acceptance in the management of small adrenal tumors and unilateral adrenal disease. This is because even individuals with a unilateral adrenal gland can potentially experience adrenal insufficiency during stressful circumstances (8). Partial adrenalectomy (PA) is associated with a significantly shorter hospital stay, shorter operative time, and fewer overall complications than total adrenalectomy (TA) (9). In cases where it is technically feasible, partial adrenalectomy (PA) may be regarded as a superior treatment option for unilateral aldosterone-producing adenoma (9). Consequently, the utilization of PA has become more prevalent in order to avoid the need for lifelong steroid replacement and to minimize the risk of recurrence, particularly in cases of bilateral adrenal diseases (7, 8, 10).

RLA, which was carried out in prone position, is widely recognized approach for treating adrenal surgical diseases in Europe (6, 11).But the ‘three-level’ RLA method, which was carried out in lateral position is more popular in mainland China over the past three decades, as reported by Zhang et al. in 2007 (12). This method simplifies and standardizes the procedures of RLA, leading to its widespread acceptance in the medical community. Using the ‘three-level’ approach, the presence of perirenal fat in the upper pole of the kidney may affect the exploration of adrenal tumors. Obesity-related factors were associated with prolonged total operative time (13). Body mass index (BMI) and tumor size are better indicators of the Mayo Adhesive Probability (MAP) score (14), which can influence the difficulty of RLA (15). Zhang et al. also mentioned, for obese and cushing patients, perirenal fat in the upper pole of the kidney should be excised first (12). To address the challenge of upper pole renal fat interference in surgical procedures for overweight and obese patients, we implemented modified three-level retroperitoneal laparoscopic techniques in patients with a BMI ≥ 25 Kg/m2, resulting in significant enhancements (16). We further generalize this technology to encompass the entire population.

## 2 Patients and methods

### 2.1 Patients

This retrospective study was performed at Fujian Provincial Hospital in Fujian Province. This is a tertiary referral, not for-profit, high-volume comprehensive center in China serving a region with approximately 41.88 million inhabitants. We included patients who had undergone adrenal surgery at our department from January 2016 to December 2022, in a retrospective manner. The patients selected different treatment groups according to their preferences, while ensuring consistency in the title and surgical expertise within our department. The optimal approach was selected unanimously by both the surgeons and the patients, resulting in two distinct groups: Zhang’s technique (ZT) or a modified variation of the technique (MT).

**Figure 1:**
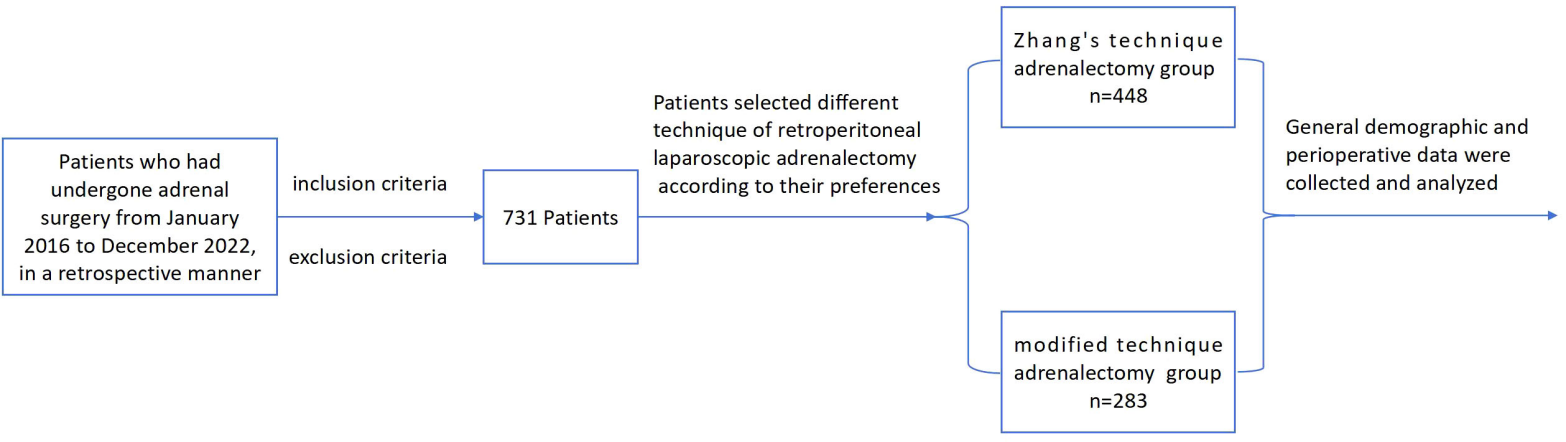
The flow-chart of a retrospective, case-control study on retroperitoneal laparoscopic adrenalectomy for all patients with adrenal lesions ≤ 6cm.

We included patients who met the following criteria: Patients who fulfilled the following criteria were included in the study: (a) Patients diagnosed with adrenal tumors or lesions who needed surgical treatment according to guideline (1) and underwent retroperitoneal laparoscopic adrenalectomy (RLA). (b) Patients who underwent preoperative CT or MRI scans of the abdomen. (c) diameter below 6 cm. The exclusion criteria for patients were as follows: (a) failure to meet the aforementioned conditions, (b) presence of adrenal malignancy, (c) recurrence of adrenal tumor postoperatively, (d) insufficient data available for analysis (**Figure 1**). This study was approved by the Ethics Committee of Fujian Provincial Hospital, Fujian Province, People’s Republic of China (approval K2023-05-008).

### 2.2 Surgical approach

**Figure 2:**
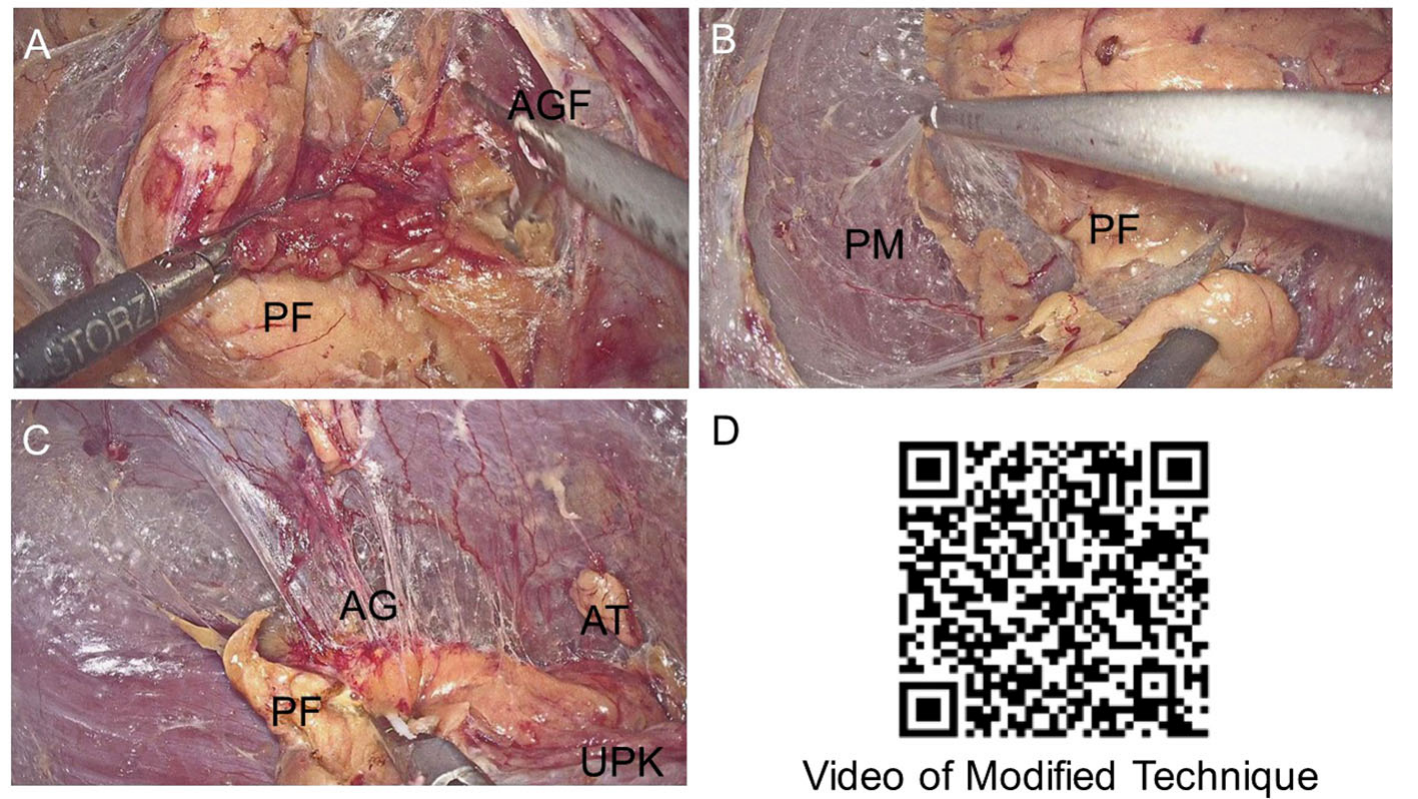
Surgical video still and surgical video. **(A)** The first separation level, the relatively avascular space between the Perirenal fat (PF) and the anterior Gerota’s fascia (AGF). **(B)** The second separation level, the relatively avascular space was between the dorsal side of PF and the psoas muscle (PM). **(C)** The modified third separation level, here latively avascular space bet we endorsalside of the adrenal gland (AG) and PF. After reaching the adrenal gland, the adrenal gland and the adenoma were exposed along the edge of the AG. Upper pole of the kidney (UPK).The video of Modified Three-Level Technique of Retroperitoneal Laparoscopic Partial Adrenalectomy was published by Chinese Journal of Endourology (Electronic Edition). **(D)** The video of Modified Three-Level Technique of Retroperitoneal Laparoscopic Partial Adrenalectomy was published by Chinese *Journal of Endourology (Electronic Edition)*.

**Figure 3:**
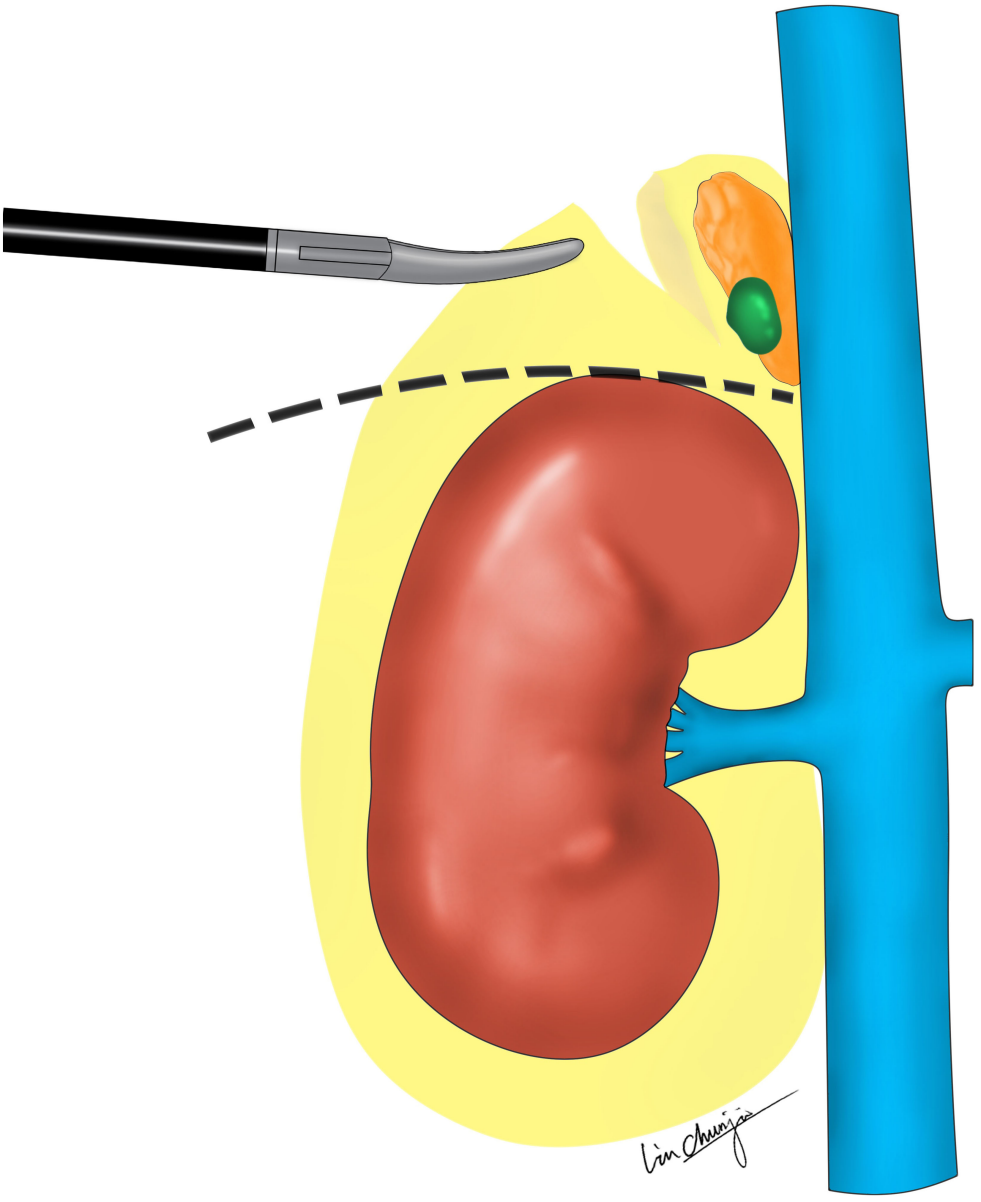
The schematic diagram of modified and three-level technique of retroperitoneal laparoscopic adrenalectomy. The modified third separation level, between dorsal side of the adrenal gland (AG) and PF. The dashed line indicates the direction of separation for the third layer of ZT. The auxiliary hand could then pull the peri-renal fat outwards and downwards to create a large enough operation space at the upper pole of the kidney. To eliminate interference from the PF and expose the AG and adenoma better, compared with ZT.

The detailed steps have been described in the previous article (16).The surgical video still was demonstrated in **Figure 2**, and the complete procedure video (17) can be accessed by scanning **Figure 2D**. The schematic diagram of modified and three-level technique of retroperitoneal laparoscopic adrenalectomy was shown on **Figure 3**.

### 2.3 Statistical analysis

Statistical analysis was conducted using R software. We calculated descriptive statistics for the variables: frequencies and percentages were computed for categorical variables, while means and standard deviations were calculated for numerical variables that exhibited a normal distribution. Analysis of variance (ANOVA) was performed on normally distributed data and is presented as mean ± standard deviation. For non-normally distributed data, the median (interquartile range) was used along with non-parametric tests, and the significance level was set at p < 0.05.

## 3 Results

### 3.1 General demographic data

A total of 731 patients were included in this study. The patients were divided into two groups based on the surgeon’s choice: ZT group (n=448) and MT group (n=283). In the ZT group, there were 201 male and 247 female participants, with an average BMI of 23.53 (21.48, 25.80) Kg/m2 and an average tumor diameter of 3.04 ± 1.25 cm. The MT group consisted of 119 male and 164 female participants, with an average BMI of 23.73 (21.65, 25.71) Kg/m2 and an average tumor diameter of 2.96 ± 1.49 cm. In the MT group, the lesions were located on the left and right sides in 173 and 110 patients, respectively. In the ZT group, 276 and 172 patients had left-sided and right-sided lesions, respectively. There were no statistically significant differences observed between the two groups regarding variables such as sex, BMI, tumor location, tumor size, tumor type, and American Society of Anesthesiologists (ASA) score(p>0.05) (18). Patients in the ZT group were marginally older than those in the MT group [ZT, 51.62 ± 11.45 years vs. MT, 48.84 ± 12.22 years p < 0.05] (**Table 1**).

**Table 1:** General demographic data of ZT group and MT group.

Characteristic	MT, N = 283	ZT, N = 448	p
Gender			0.763
Male	119 (42%)	201(45%)	
Female	164 (58%)	247 (55%)	
Age(years)	48.84 ± 12.22	51.62 ± 11.45	0.001
BMI	23.53 (21.48, 25.80)	23.73 (21.65, 25.71)	0.615
Tumor location			0.910
Right	110 (39%)	172 (38%)	
Left	173 (61%)	276 (62%)	
Tumour size (cm)	2.96 ± 1.49	3.04 ± 1.25	0.243
ASA score			0.758
I	1 (0.4%)	5 (1.1%)	
II	178 (63%)	277 (63%)	
III	103 (36%)	160 (36%)	
IV	1 (0.4%)	1 (0.2%)	
Tumor type (n)			0.662
Adrenocortical adenoma	208 (73%)	338 (75%)	
Pheochromocytoma	29 (10%)	36 (8.0%)	
Others	46 (16%)	74 (17%)	

ZT, Zhang’s technique; MT, modified technique.

### 3.2 Comparison of peri-operative period between the two groups

All of the patients underwent endoscopic surgery without any conversions to open surgery, indicating successful outcomes for all cases. The MT group demonstrated superior outcomes compared to the ZT group in terms of operative time, estimated blood loss, drainage, diet recovery, complication rates, and postoperative hospitalizations postoperative hospitalization duration (p<0.05) (**Table 2**). In the MT group, 256 of the 283 patients underwent planned adrenal-sparing surgery without requiring unplanned adrenalectomy. Conversely, in the ZT group, among 392 (87.5%) of the 448 patients who were planned for adrenal-sparing surgery, 17 (4.34%) required unplanned adrenalectomy (P<0.05) (**Table 3**).

**Table 2:** Comparison of perioperative period between ZT group and MT group.

Perioperative data	MT, N = 283	ZT, N = 448	p
Operative time* (min)	98.26 ± 36.95	109.42 ± 43.22	0.000
Estimated blood loss (ml)	22.48 ± 27.06	32.64 ± 74.27	0.002
Drainage (days)	2.69 ± 1.05	4.29 ± 2.69	0.000
Diet recovery (days)	1.04 ± 0.20	1.86 ± 0.85	0.000
Complications rates (n)	18 (6.4%)	51 (11%)	0.024
Clavien-Dindo			0.000
0	266 (94%)	406 (91%)	
I	6 (2.1%)	0 (0%)	
II	10 (3.5%)	37 (8.3%)	
III	1 (0.4%)	2 (0.4%)	
IV	0 (0%)	3 (0.7%)	
Postoperative hospitalizations duration (days)	3.62 ± 1.43	6.19 ± 2.36	0.000

*The operation time was defined as the duration between the initiation and termination of anesthesia.

**Table 3:** The rates of unplanned adrenalectomy between ZT group and MT group.

	Planned adrenalectomy	Planned adrenal sparing	*Unplanned adrenalectomy	p
ZT	56	392	17 (4.34%)	0.000
MT	27	256	0	

*Unplanned adrenalectomy was defined as an attempt to spare the adrenal gland, but due to inadequate exposure, a total adrenalectomy was performed.

## 4 Discussion

In our retrospective study, according to the surgeon’s decision, all patients were divided into the ZT and MT groups. Patients in the ZT group were marginally older than those in the MT group, but no statistically significant differences were observed in the ASA scores between the two groups. The MT group exhibited superior postoperative outcomes compared with the ZT group. In addition, unplanned adrenalectomy was not required in the MT group, whereas 17 patients (4.34%) in the ZT group required unplanned adrenalectomy. The aforementioned data analysis indicated that MT retroperitoneal laparoscopic adrenalectomy was beneficial for the treatment of adrenal lesions in all the patients.

Minimally invasive adrenalectomy has been associated with excellent outcomes (19). Analysis of data from the EUROCRINE database provides support for the assertion that robotic adrenalectomy (RA) is associated with a lower complication rate and shorter hospital stays compared to laparoscopic adrenalectomy (20), and excellent functional results (21). However, because of its significantly higher cost (22), RA may not be a suitable option for everyone in China or other developing countries. Laparoscopic adrenalectomy (LA) is an accepted treatment for adrenal gland diseases in adults (23). Over the past two decades, there has been a consistent rise in interest regarding partial adrenalectomy (PA) (6). In a unilateral primary aldosteronism (uPHA) setting, minimally invasive partial adrenalectomy (MIPA) showed excellent perioperative results with a complete clinical success rate of 72.4% (24). In other study, PA and TA provide comparable perioperative and functional outcomes (25).Adrenal pathologies are mostly benign, making organ-preserving procedures attractive for many patients (6).

Some modified techniques have been implemented with the aim of reducing operative time, achieving significant cosmetic benefits, and minimizing incisional pain (26, 27), none of them mentioned unplanned adrenalectomy. Unplanned adrenalectomy was defined as an attempt to spare the adrenal gland; however, due to inadequate exposure or other reasons, total adrenalectomy was performed. In our study, 17 patients (4.34%) in the ZT group required unplanned adrenalectomy, which was significantly different from the MT group, where no such cases occurred. Although not all patients underwent total adrenalectomy requiring oral corticosteroids, it was advisable to preserve the adrenal gland based on preoperative physician assessment.

The overall operation time of the MT group was 98.26 ± 36.95 minutes. As this was a retrospective study, and video recordings were not available for each operation, the precise duration from skin resection to skin suturing could not be obtained. Therefore, we defined the operative time as the period between anesthesia induction and emergence generated by the anesthesia system. In a retrospective observational study of 580 patients, divided into RLA approaches and TLA approaches, with respective operation times of 117.02 ± 54.98 minutes and 83.65 ± 31.22 minutes. The overall duration of surgery was comparable to that in our own investigation. In a large study of laparoscopic adrenalectomy, 3946 patients who were categorized based on their non-functional adrenal adenoma, functional seboradenoma, pheochromocytoma and aldosteronoma, the operation time for these groups was found to be 127.8 ± 58.9 min, 168.5 ± 106.0 min, 134.9 ± 54.2 min and 120.8 ± 53.9 min respectively, which is longer than our operation time (28). Therefore, in terms of operating time, our MT can be considered similar to or better than conventional laparoscopic adrenalectomy.

​The incidence of complications is an essential indicator for evaluating the merits and disadvantages of the surgical techniques. According to the Clavien-Dindo classification, 51 cases (11%) in the ZT group had postoperative complications, which is comparable to a study of 1,005 patients with an overall complication rate of 13.7% (20). In contrast, the MT group had a lower rate of postoperative complications, with only 18 (6.4%) patients experiencing complications. This was lower than that in the ZT group and other reported rates. Only one patient in the MT group had Clavien-Dindo III complications, and cystoscopy was necessary to confirm the diagnosis of painless hematuria. Therefore, MT appears to be a safe approach to treat RLA.

Our study showed that within the MT group, the estimated blood loss was 22.48 ± 27.06 ml, the duration of drainage was 2.69 ± 1.05 days, the time for postoperative diet recovery was 1.04 ± 0.20 days, and the length of postoperative hospital stay was 3.62 ± 1.43 days. These results indicate a better postoperative outcome than that in the ZT group. Despite the small difference in estimated blood loss, the large sample size in our study resulted in a statistically significant difference between the two groups. At our institution, patients are typically discharged the day after drainage tube removal, for safety reasons and customary practices. It should be noted that although the MT group had a shorter surgical time than the ZT group, which may result in better outcomes, there is still a possibility that the difference in the time for drainage tube removal and postoperative diet recovery may be due to differences in physician practices. Although all surgeons received the same surgical technique training, the postoperative management was not uniform or double-blinded within the department. Additionally, our study did not analyze the correlation between tumor type and surgical method in terms of postoperative blood pressure control. But there have been some excellent studies showing that the therapeutic intensity score (TIS) and adenoma size (AS) can serve as valuable indicators in identifying patients with unilateral primary aldosteronism (UPA) who are at a higher risk of experiencing persistent hypertension following either TA or PA (29). The Trifecta, defined as a ≥50% reduction in antihypertensive therapeutic intensity score (ΔTIS), absence of hypokalemia at 3 months, and no Clavien grade 2–5 complications,and AS, independently predict long-term complete clinical, biochemical, or combined success after adrenalectomy for UPA (30).

A limitation of this study is that it was a retrospective study conducted at our institution. Although previous studies have shown the advantages of the MT procedure (16), other surgeons with comparable experience may perceive the difference to be insignificant because of the significant differences in sample sizes between the two groups. Stratified analyses for patients with different MAP scores have not been conducted at this time (14) and adrenal lesion locations, which is our next step. We hope that further studies, especially randomized studies, will evaluate the implementation of this improved technique in more medical centers in the future, and this technology will be generalizable to different patient populations or different healthcare Settings.

## 5 Conclusion

MT retroperitoneal laparoscopic adrenalectomy has demonstrated its benefits in the treatment of adrenal lesions across all patients, serving as a valuable point of reference for the surgical management of adrenal diseases.

## Data availability statement

The raw data supporting the conclusions of this article will be made available by the authors, without undue reservation.

## Ethics statement

This study was approved by the Ethics Committee of Fujian Provincial Hospital, Fujian Province, People’s Republic of China (approval K2023-05-008). The studies were conducted in accordance with the local legislation and institutional requirements. The participants provided their written informed consent to participate in this study. Written informed consent was obtained from the individual(s) for the publication of any potentially identifiable images or data included in this article.

## Author contributions

MH: Conceptualization, Funding acquisition, Writing – original draft. YW: Data curation, Formal analysis, Writing – review & editing. XX: Investigation, Writing – review & editing. WCC: Investigation, Writing – review & editing. JL: Validation, Writing – review & editing. WHC: Investigation, Writing – review & editing. HP: Investigation, Writing – review & editing. ZY: Formal analysis, Software, Writing – review & editing. LY: Conceptualization, Funding acquisition, Writing – review & editing. JW: Conceptualization, Funding acquisition, Writing – review & editing.
